# Prospective comparative study of tolerance to refractive errors after implantation of extended depth of focus and monofocal intraocular lenses with identical aspheric platform in Korean population

**DOI:** 10.1186/s12886-019-1193-z

**Published:** 2019-08-19

**Authors:** Hyeck-Soo Son, Seong Ho Kim, Gerd U. Auffarth, Chul Young Choi

**Affiliations:** 1 International Vision Correction Research Centre (IVCRC), Department of Ophthalmology, University of Heidelberg, Heidelberg, Germany; 2 Department of Ophthalmology, Kangbuk Samsung Hospital, Sungkyunkwan University School of Medicine, 29 Saemunan-ro, Jongno-gu, Seoul, 03181 Republic of Korea

**Keywords:** Extended depth of focus IOL, Symfony, ZXR00, Visual acuity tolerance to postoperative refractive errors

## Abstract

**Background:**

To evaluate the clinical outcomes of extended depth of focus (EDOF) and monofocal intraocular lenses (IOLs) that share identical aspheric platform and compare their visual acuity tolerance to postoperative refractive errors.

**Methods:**

This non-randomized, prospective comparative study included 120 eyes undergoing cataract surgery with implantation of either Tecnis ZCB00 IOL (Abbott Medical Optics Inc., Santa Ana, CA) (monofocal group: 60 eyes of 30 patients) or Tecnis Symfony IOL (Abbott Medical Optics, Inc.) (EDOF group: 60 eyes of 30 patients). Monocular and binocular visual outcomes, changes in refraction, defocus curve, contrast sensitivity, and perception of photic phenomena (Halo & Glare Simulator; Eyeland Design Network, Vreden, Germany) were evaluated 3 months postoperatively. To compare the refractive tolerance, each group was divided into three subgroups according to the postoperative uncorrected distance visual acuity (UDVA) and postoperative spherical equivalent (SE).

**Results:**

In the EDOF group, the mean 3-months postoperative monocular UDVA, intermediate (UIVA), and near (UNVA) visual acuities were 0.03 ± 0.07, 0.09 ± 0.15, and 0.24 ± 0.16 logMAR, respectively. A total of 100, 96.55, and 68.97% of eyes in the EDOF group achieved binocular UDVA, UIVA, and UNVA values of 0.20 logMAR or better, respectively. In respect to refractive tolerance, the EDOF group showed higher SE values and statistically significantly better mean UDVA than the monofocal group in all subgroups, with UDVA of − 0.013 and 0.028 logMAR for EDOF and monofocal groups (*p* = 0.037), respectively, in the subgroup where SE was within ±0.50 D, UDVA of 0.004 and 0.048 logMAR for EDOF and monofocal groups (*p* = 0.046), respectively, in the subgroup where SE was within − 1.00 D, and UDVA of 0.020 and 0.083 logMAR for EDOF and monofocal groups (*p* = 0.026), respectively, in the subgroup where SE was more than − 1.00 D. The mean patient satisfaction scores for spectacle-free distance, intermediate, and near visual acuities were 86.0, 85.0, and 66.0, respectively.

**Conclusions:**

The EDOF IOL provided excellent postoperative visual outcomes in far and intermediate distances, with high patient satisfaction rate. Regarding the postoperative refractive tolerance to SE, the Tecnis Symfony IOL showed better tolerance to residual postoperative refractive error than the monofocal IOL with the same material and optical platform.

## Background

Today, multifocal intraocular lenses (IOLs) seem to offer the most promising treatment option for presbyopic patients [1]. Despite numerous advantages of multifocal IOLs, factors such as residual refractive error can lead to high dissatisfaction rate [2]. Minimum postoperative refractive error is required to achieve optimal visual outcomes, with even minor levels of astigmatism significantly undermining the patients’ postoperative visual acuity [3]. Refractive error, therefore, needs to be corrected as much as possible in order to fully exploit the benefits of multifocal IOLs [4]. The estimated percentage of enhancement procedures performed to reduce residual astigmatism after implantation of multifocal lenses varies from 5.24 to 23.66% depending on the study. For example, Gundersen et al. [5] observed considerable retreatment rates (10.8%), most of which were due to decrease in visual acuity (VA) secondary to residual astigmatism.

An extended depth of focus (EDOF) IOL provides significantly increased range of vision with minimal optical side effects of multifocality [6, 7]. Currently, there are no studies comparing the refractive error tolerance of an EDOF IOL to that of a monofocal one. Therefore, the aim of this study was to assess the clinical performance of an EDOF IOL (Tecnis® Symfony ZXR00) and compare its visual acuity tolerance to postoperative refractive errors of a monofocal IOL (Tecnis® ZCB00).

### Patient and methods

In this prospective comparative study, 60 patients who underwent cataract surgery with implantation of either EDOF IOLs or monofocal IOLs that share the same material and aspheric platform were included. The EDOF group included 30 patients with bilateral implantation of the Tecnis® Symfony IOL (Abbott Medical Optics, Inc.), while the monofocal group included 30 patients with bilateral implantation of the aspheric Tecnis® ZCB00 IOL (Abbott Medical Optics, Inc.). Every patient was informed about the inclusion in the study and provided a written consent. The study procedure complied with the tenets of the Declaration of Helsinki and was approved by the local institutional review board.

Inclusion criteria consisted of patients with cataract and preexisting corneal astigmatism of 1.25 diopters (D) or less. Patients with a history of ocular pathology, trauma, contact lens wear, pregnancy, systemic or local medication, and ocular surgeries other than laser refractive surgery for myopia were excluded.

### Examination protocol

Preoperative ophthalmological examination included manifest refraction, monocular corrected distance visual acuity (CDVA), Goldmann applanation tonometry, slit-lamp examination, corneal topography (Galilei G6; Ziemer Ophthalmic Systems AG, Port, Switzerland), optical biometry (IOLMaster 500; Carl Zeiss Meditec, Jena, Germany), funduscopy, and pupil size measurement (KR-1 W, Wavefront Analyser; Topcon, Tokyo, Japan)).

Postoperative follow-up examinations were performed 1 day after surgery as well as 1 week, 1 month, and 3 months postoperatively. The postoperative examination at 1 month and 3 months included measurements of monocular and binocular uncorrected distance visual acuity (UDVA), CDVA, uncorrected intermediate visual acuity (UIVA) at 66 cm, binocular near visual acuity (UNVA) at 40 cm, contrast sensitivity under photopic (85 cd/m^2^) and mesopic conditions (3 cd/m^2^) (CSV-1000, VectorVision, Greenville, OH), and halo and glare using a computer-based software (Halo & Glare Simulator; Carl Zeiss Meditec). The simulator utilizes a numerical scale to quantify the size and intensity of halos and glare, ranging from 0 (none), 25 (mild), 50 (moderate), 75 (severe) to 100 (very severe). The simulator also classifies the halos perceived by patients into three different types: T1 (diffuse halo ring), T2 (starburst type), and T3 (distinct halo ring). In addition, at 3 months postoperatively, the pupil size was measured again using the same measurement device as noted above.

All patients were asked about their usage of spectacles after surgery with the question: “How often do you need spectacles to see at far/intermediate/near distances?” The answer choices were classified as 0, 25, 50, 75, and 100% of time. Furthermore, patients were asked about their satisfaction with the postoperative results with: “How satisfied are you with your spectacle-free vision at far/intermediate/near distances?” The answer choices ranged from 0 (not at all satisfied) to 100 (very satisfied). In addition, they were asked following questions: “Would you choose the same lens again?” and “Would you recommend this lens to your relatives and friends?”

Uncorrected monocular and binocular defocus curves were recorded from + 1.00 D to − 4.00 D in 0.50-D steps. All data were computed into a Cartesian graphic display, with the x-axis indicating the level of defocus and the y-axis the visual acuity values.

For comparison of the visual acuity tolerance to postoperative refractive errors in EDOF and monofocal IOL groups, each IOL group was subdivided into three groups depending on the measured postoperative uncorrected distance visual acuity: less than 0.0 (1.0 in decimal), 0.1 (0.8 in decimal), and more than 0.2 logMAR (0.63 in decimal). Parallelly, another subdivision was made in which each IOL group was divided according to the achieved postoperative spherical equivalent (SE) values: within ±0.50 D, within − 1.00 D, and larger than − 1.00 D.

### Surgical procedure

One experienced surgeon (C.Y.C.) performed all surgeries using standard phacoemulsification via sutureless 2.2-mm incision. All incisions were placed at the steepest corneal meridian and topical anesthesia as well as mydriatic drops were instilled prior to the surgical procedure. After performing capsulorhexis and phacoemulsification, the study IOL was placed into the capsular bag using the Unfolder Platinum 1 series screw-style inserter (Abbott Laboratories, Inc.) through the main incision. In both groups, emmetropia or minimal myopia was aimed as target refraction in IOL power calculation using the Haigis formula.

### Statistical analysis

A statistical software SPSS (Version 24.0 for Windows; IBM, Armonk, NY) was used for statistical analysis. The Kolmogorov–Smirnov test was used to check the normality of the data distribution. When parametric analysis was possible, the Student’s t test for paired data was performed for parameter comparisons between preoperative and postoperative results. In cases when parametric analysis was not possible, the Mann-Whitney U test was applied to assess the significance of differences between the results. An independent two sample T-test was performed for statistical analysis of pupil size comparison between the two groups. In all cases, *P* value < 0.05 was considered as statistically significant.

## Results

The analysis included 60 patients who completed the 3-month follow-up examinations. The results for the 3-month timeline were stratified based on the available patients in this as well as patients with EDOF IOLs (EDOF group) and patients with monofocal IOLs in both eyes (monofocal group). Table 1 gives an overview of the patients’ demographics. There were no significant differences in preoperative visual acuity values between the monofocal and the EDOF IOL groups.

**Table 1:** Patients demographics

Parameter	EDOF group	Monofocal group	*P* value
Eyes, n	58	60	
Patients, n	29	30	
Age (years)			0.283
Mean ± SD	64.59 ± 10.00	66.35 ± 8.71	
Range	45 to 84	48 to 82	
Male gender, n (%)	10 (34.5)	14 (46.7)	0.192

*D* diopters, *EDOF* Extended depth of focus, *SD* standard deviation, *SE* spherical equivalent

### Visual and refractive outcomes and spectacle independence

Table 2 demonstrates the preoperative and postoperative visual outcomes of all patients. Overall, statistically significant improvements in monocular and binocular CDVA, UDVA, UIVA and UNVA were found after surgery (*P* <  0.001). When a comparison was made between monocular and binocular visual acuities at 3-months postoperatively, binocular CDVA (*P* = 0.020) and UDVA (*P* = 0.005) showed statistically significant superiority to monocular CDVA and UDVA. However, there were no statistically significant differences between binocular and monocular UIVA (*P* = 0.174) and between binocular and monocular UNVA (*P* = 0.066). Changes in refractive sphere (*P* <  0.001) and cylinder (*P* = 0.017) values also reached statistical significance at 3 months postoperatively, while changes in spherical equivalent (*P* = 0.253) did not.

**Table 2:** Preoperative and postoperative 3 months visual outcomes of EDOF group

Variable	Preoperative	Monocular	Binocular	*P* value^†^
UDVA (logMAR)				< 0.001^*^
Mean ± SD	0.46 ± 0.43	0.03 ± 0.07	−0.01 ± 0.05	
Range	0 to 1.6	−0.1 to 0.3	−0.1 to 0.1	
CDVA (logMAR)				< 0.001^*^
Mean ± SD	0.21 ± 0.31	−0.02 ± 0.07	−0.06 ± 0.06	
Range	−0.1 to 1.2	−0.2 to 0.2	− 0.2 to 0.1	
UIVA (logMAR)				< 0.001^*^
Mean ± SD	0.60 ± 0.36	0.09 ± 0.15	0.04 ± 0.13	
Range	0.1 to 1.3	−0.1 to 0.6	−0.1 to 0.5	
UNVA (logMAR)				< 0.001^*^
Mean ± SD	0.70 ± 0.37	0.24 ± 0.16	0.17 ± 0.14	
Range	0.1 to 2.0	−0.1 to 0.5	− 0.1 to 0.4	

*CDVA* corrected distance visual acuity, *D* diopters, *DCIVA* distance corrected intermediate visual acuity, *EDOF* Extended depth of focus, *SD* standard deviation, *UDVA* uncorrected distance visual acuity, *UIVA* uncorrected intermediate visual acuity, *UNVA* uncorrected near visual acuity

^†^For monocular comparison

^*^P value is statistically significant (*P* <  0.05)

Table 3 summarizes the postoperative monocular visual and refractive data of the two groups. As shown, the EDOF group achieved significantly better CDVA than the monofocal group (*P* = 0.008). Monocular UDVA of 0.20 logMAR or better was found in 98 and 96% of eyes in the EDOF and the monofocal group, respectively. Mean postoperative spherical equivalent was − 0.81 D (− 1.75 to + 0.50 D) and − 0.40 D (− 1.50 to + 0.25 D) in the EDOF and the monofocal group, respectively (*P* <  0.001), and postoperative spherical equivalent was within ±1.00 D in 82.5 and 90.8% of eyes in the EDOF and the monofocal groups, respectively. Mean postoperative cylinder was − 0.59 D (− 1.75 to 0.00 D) and − 0.58 D (− 1.75 to 0.00 D) in the EDOF and the monofocal group, respectively (*P* = 0.896).

**Table 3:** Comparison of postoperative visual acuity and refractive data

Variable	EDOF IOL (58 eyes)	Monofocal IOL (60 eyes)	*P* value
UDVA (logMAR)			0.530
Mean ± SD	0.03 ± 0.07	0.06 ± 0.09	
Range	−0.1 to 0.3	0 to 0.3	
CDVA (logMAR)			0.008^*^
Mean ± SD	−0.02 ± 0.07	0.01 ± 0.03	
Range	−0.2 to 0.2	0.0 to 0.1	
Sphere (D)			0.033^*^
Mean ± SD	−0.53 ± 0.42	−0.25 ± 0.50	
Range	−1.75 to 0.50	− 1.25 to 0.50	
Cylinder (D)			0.896
Mean ± SD	−0.59 ± 0.48	−0.58 ± 0.45	
Range	−1.25 to 0.00	−1.25 to 0.00	
SE (D)			< 0.001^*^
Mean ± SD Range	−0.81 ± 0.40 − 1.75 to 0.50	− 0.40 ± 0.40 − 1.50 to 0.25	
Pupil Size (mm)			0.270
Mean ± SD	3.86 ± 0.45	3.76 ± 0.56	

*CDVA* corrected distance visual acuity, *D* diopters, *EDOF* extended depth of vision, *IOL* intraocular lens, *SD* standard deviation, *SE* spherical equivalent, *UDVA* uncorrected distance visual acuity

^*^P value is statistically significant (*P* < 0.05)

**Fig. 1:**
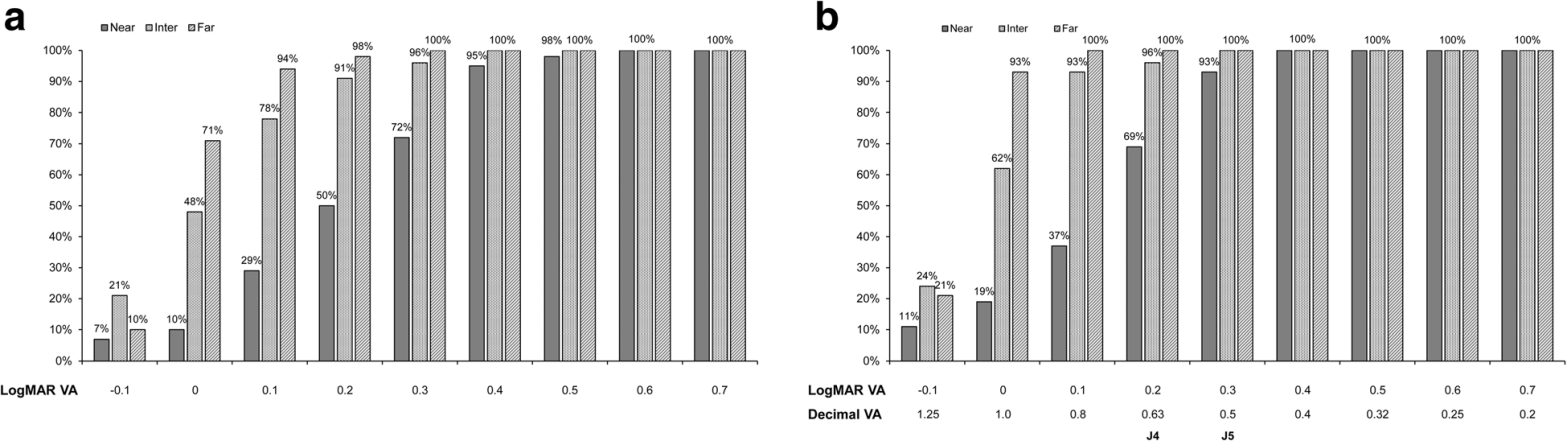
Distribution of 3-month postoperative (**a**) monocular and (**b**) binocular uncorrected visual acuity of EDOF IOL group

Figure 1 demonstrates the distribution of 3-month postoperative monocular and binocular visual acuity values in the EDOF group. A total of 98.28, 91.38, and 50.00% of eyes achieved monocular UDVA, UIVA, and UNVA of 0.20 logMAR or better. Binocularly, UDVA, UIVA, and UNVA achieved values of 0.20 logMAR or better in 100.00, 96.55, and 68.97% of eyes, respectively. And more than 90% of all patients achieved 0.40 logMAR and 0.5 logMAR in monocular and binocular UNVA, respectively. Binocular postoperative CDVA values were 0.10 logMAR or better in all cases.

The level of spectacle independence reported by patients is demonstrated in Table 4. It shows that 96, 92 and 75% of patients with the EDOF IOL never or only occasionally requires spectacles for distance, intermediate, and near vision, respectively.

**Table 4:** Postoperative spectacle independence data 3 months after surgery in EDOF group

Spectacle independence	Percent (%)
Distance (%)	
Never/occasionally	96
50% of time	4
Frequently	0
Intermediate (%)	
Never/occasionally	92
50% of time	8
Frequently	0
Near (%)	
Never/occasionally	75
50% of time	14
Frequently	11

*EDOF* extended depth of focus

### Defocus curve outcomes

**Fig. 2:**
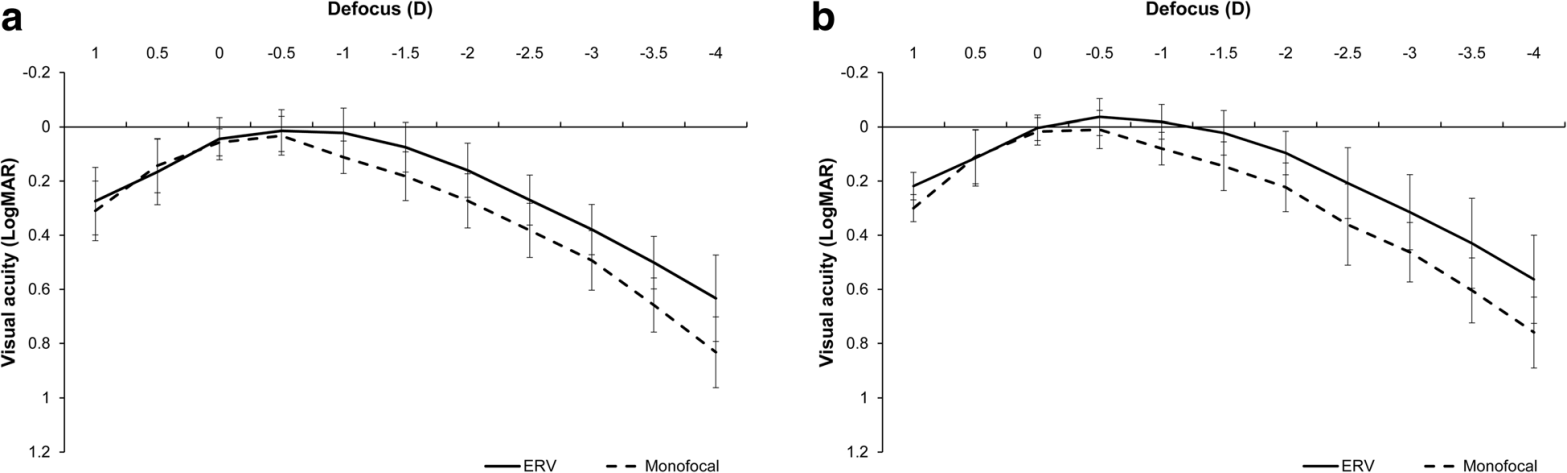
Mean (**a**) monocular and (**b**) binocular defocus curves obtained in the extended depth of focus (EDOF) and monofocal groups

Figure 2 shows the mean monocular and binocular defocus curve results of the two groups. Under monocular and binocular conditions, no significant differences were found between the two groups for the defocus levels of − 0.50 D (monocular, *P* = 0.298; binocular, *P* = 0.978), 0.00D (monocular, *P* = 0.530; binocular, *P* = 0.874), and + 0.50 D (monocular, *P* = 0.502; binocular, *P* = 0.578). For the rest of the defocus levels, monocular and binocular visual acuity values were superior in the EDOF group (*P* ≤ 0.05).

### Photic phenomena outcomes

In EDOF group, halos were reported by 11 patients (37%) and glare by 4 patients (13%) (Table 5). 14 patients (47%) indicated to experience no or mild levels of halos, glare, starbursts, and other types of dysphotopsia. At the 3-month postoperative assessment, 5 patients (16%) reported to experience severe visual symptoms. In contrast, in monofocal group, there were much less patients that experienced halos, glare, starbursts, and other types of photic phenomena (*P* = 0.956, *P* = 0.557, *P* = 0.046, and *P* = 1.000, respectively).

**Table 5:** Incidence and level of photic phenomena 3 months after surgery

Photic phenomenon	EDOF group	Monofocal group	*P* value
Type 1 Halo			0.956
No	28 (94)	29 (97)	
Mild	1 (3)	1 (3)	
Moderate	1 (3)	0	
Severe	0	0	
Very severe	0	0	
Type 2 Halo			0.046^*^
No	24 (70)	28 (94)	
Mild	3 (10)	1 (3)	
Moderate	2 (7)	1 (3)	
Severe	3 (10)	0	
Very severe	1 (3)	0	
Type 3 Halo			1.000
No	30 (100)	30 (100)	
Mild	0	0	
Moderate	0	0	
Severe	0	0	
Very severe	0	0	
Glare			0.557
No	26 (87)	28 (93)	
Mild	2 (7)	2 (7)	
Moderate	1 (3)	0	
Severe	1 (3)	0	
Very severe	0	0	

Data are presented as number (%)

Type 1, 2, 3 Halo represent diffuse halo ring, starburst type, and distinct halo ring, respectively

*EDOF* extended depth of focus

^*^P value is statistically significant (*P* < 0.05)

### Contrast sensitivity outcomes

**Fig. 3:**
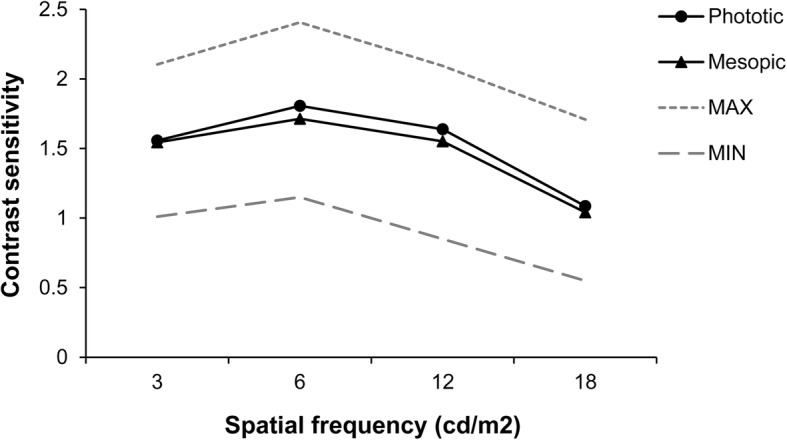
Mean contrast sensitivity function of EDOF IOL group under mesopic (gray line) and photopic (black line) conditions at 3 months postoperatively. The results are also compared with the ranges of normality defined previously for the contrast sensitivity test used (Data from Pomerance G, Evans D. Test-retest reliability of the CSV-1000 contrast test and its Vis Sci. 1994; 35: 3357–3361)

Figure 3 shows the binocular contrast sensitivity results of the EDOF group under photopic and mesopic conditions. At all spatial frequencies, binocular contrast sensitivity values were within the normal ranges reported by the CSV-1000 [8].

### Tolerance to postoperative residual refractive errors

In subgroups consisting of patients with postoperative UDVA of 0.1 logMAR or postoperative UDVA of less than 0.0 logMAR, EDOF group had significantly myopic spherical equivalent than the monofocal group (*P* = 0.049 and *P* = 0.039, respectively) (Table 6).

**Table 6:** Correlations of UDVA and postoperative refractive errors

Variable	EDOF IOL (D)	Monofocal IOL (D)	*P* value
Postoperative UDVA ≤0.0 (LogMAR, 1.0 in decimal)			0.049^*^
Sph	−0.46	−0.15	
Cyl	−0.52	−0.53	
SE	−0.71	−0.36	
Postop UDVA = 0.1 (LogMAR, 0.8 in decimal)			0.039^*^
Sph	−0.69	−0.10	
Cyl	−0.57	− 0.91	
SE	−1.05	−0.59	
Postop UDVA ≥0.2 (LogMAR, 0.63 in decimal)			0.916
Sph	−0.71	−0.70	
Cyl	−1.06	−1.05	
SE	−1.26	−1.25	

*EDOF* extended depth of focus, *IOL* intraocular lens, l*ogMAR* logarithm of the minimum angle of resolution, *SE* spherical equivalent, *UDVA* uncorrected distance visual acuity

^*^P value is statistically significant (*P* < 0.05)

When the subgroups were divided according to the postoperative refractive errors, all EDOF subgroups had significantly better mean UDVA than the monofocal subgroups (*P* = 0.037, *P* = 0.046 and *P* = 0.026, respectively) (Table 7).

**Table 7:** Influence of residual spherical equivalent on visual acuity

Post-operative spherical equivalent	EDOF IOL, Decimal (logMAR)	Monofocal IOL, Decimal (logMAR)	*P* value
Within ±0.50 D	1.04 (− 0.013)	0.94 (0.028)	0.037^*^
Within − 1.00 D	0.99 (0.004)	0.91 (0.048)	0.046^*^
More than − 1.00 D	0.96 (0.020)	0.84 (0.083)	0.026^*^

*EDOF* extended depth of focus, *IOL* intraocular lens, *logMAR* logarithm of the minimum angle of resolution, *SE* spherical equivalent, *UDVA* uncorrected distance visual acuity, *VA* visual acuity

^*^P value is statistically significant (*P* < 0.05)

### Pupil size

The mean preoperative pupil sizes were 3.92 ± 0.53 mm and 3.90 ± 0.49 mm in the EDOF and monofocal group, respectively. At 3 months postoperatively, the mean pupil sizes were 3.86 ± 0.45 mm and 3.76 ± 0.56 mm in the EDOF and monofocal group, respectively. In both pre- (*p* = 0.779) and postoperative (*p* = 0.270) measurements, there were no statistically significant differences in the mean pupil sizes between the two groups.

### Patient satisfaction

The mean patient satisfaction scores for distance, intermediate, and near visual acuities were 86.0, 85.0, and 66.0, respectively. 27 patients (93.1%) stated that they would recommend the same treatment to their friends and family. Also, 26 patients (89.6%) said they would choose the same IOL again.

### Complications

One eye (1.7%) in EDOF group and three eyes (5.0%) in monofocal group developed posterior capsule opacification which required neodymium: YAG capsulotomy. No complication led to IOL explantation.

## Discussion

Multifocal IOLs can successfully restore both near and distance visual acuities and yield satisfactory outcomes in the majority of patients [9, 10]. Such design can offer both cataract and presbyopic treatment, but bears disadvantages that are ascribable to its inherent optical design such as perception of photic phenomena, reduced contrast sensitivity, and decreased visual function in dim light settings [11–16]. Furthermore, residual refractive error can considerably undermine the postoperative visual performance, causing high dissatisfaction rate [2].

An EDOF IOL constitutes the most recent form of multifocal technology and has been reported to provide significantly increased range of vision with minimal optical side effects [6, 7]. In previous studies, the EDOF IOLs were able to restore excellent far and intermediate visual acuity with functional near vision compared to other multifocal IOL designs [17–21]. Furthermore, the EDOF IOLs demonstrated superior range of vision and spectacle independence than the monofocal lenses that were targeted to achieve emmetropia [22]. However, in a recent study, Cochener et al. [23] reported that while both trifocal and EDOF IOLs provided good visual acuity at all distances, near vision was statistically better in the trifocal lenses compared to the EDOF ones. The current study evaluated the clinical performance of an EDOF IOL and compared its visual acuity tolerance to postoperative refractive error to that of a monofocal IOL that uses the same aberration-correcting optical platform.

In our study, distance visual outcomes were excellent in all EDOF patients, with mean binocular UDVA and CDVA values of − 0.01 ± 0.05 logMAR and − 0.06 ± 0.06 logMAR, respectively. This confirmed the ability of the EDOF IOL to successfully restore distance visual function, as it has also been reported for other models of multifocal IOLs [16, 24–39]. The logMAR postoperative monocular and binocular UDVA of 0.00 or better was achieved by 70.69 and 93.10% of eyes in the EDOF group, respectively. These results are similar to [23, 40] or even better than the outcomes observed in previously published data on other types of multifocal lenses [29, 41, 42]. As expected, the visual outcomes for intermediate vision was also excellent, with mean monocular and binocular UIVA of 0.09 ± 0.15 and 0.04 ± 0.13, respectively. The logMAR postoperative monocular and binocular UIVA of 0.20 or better was achieved by 91.38 and 96.55% of eyes in the EDOF group, respectively, which again, were comparable or superior to those reported for other models of refractive and diffractive multifocal IOLs [16, 24–39]. Similar to previous studies [19, 23], the performance of the EDOF IOL for distance or intermediate vision was better than for near vision. The noted differences in CDVA, UDVA, UIVA, and UNVA among these studies may be ascribable to factors such as variances in visual acuity measurement methods, residual refractive errors, and study populations.

In our study, the excellent visual outcomes at far and intermediate distances were consistent with the high levels of spectacle independency. In the EDOF group, about 90% of the patients indicated to not at all need spectacles or only occasionally need them for performing tasks at far and intermediate distances. For near vision, 10.3% of patients reported to require spectacles frequently. According to the Concerto study, micro-monovision method (− 0.50 to − 0.75 residual myopia in the nondominant eye) improved UDVA, spectacle independence as well as satisfaction rate for near vision [43]. Such low levels of spectacle dependence are comparable to those reported for other multifocal lenses [33, 39]. Law et al. [33] found that a limited number of patients with trifocal IOLs experienced difficulties in performing near and intermediate visual tasks such as reading newspapers or working with a computer. In another study that assessed a different trifocal lens model, Kohnen et al. [31]^31^ noted that 100% of the patients were independent of spectacles for distance and intermediate vision, while 12% occasionally required correction for near-vision. After implantation of another trifocal IOL that combines bifocal diffractive profiles, Jonker et al. [30] found that 80% of patients were independent from spectacles.

Our dysphotopsia assessment showed that starburst was the most commonly perceived optical phenomenon (reported by 38% of patients), followed by halo (8% of patients) and glare (7% of patients). In multifocal IOLs, one image is in-focus, while the out-of-focus image is neuronally suppressed (simultaneous vision) yet still produces such unwanted dysphotopsia [44]. In contrast, the Tecnis Symfony IOL provided consistent and excellent visual acuity at all distances, with minimal levels of visual disturbances compared to other multifocal IOLs [43, 45]. Halos are not less expected with such lens design as it generates an elongated focal depth rather than one or more fixed foci. In other studies reporting bilateral implantation of multifocal IOLs, 25 to 60% of patients reported difficulties due to perception of photic phenomena postoperatively [31, 33, 34]. In this study, a computer-based halo and glare simulator was implemented for assessment of occurrence of photic phenomena in order to avoid suggestive triggering of patients’ answers. As explained earlier, the Tecnis Symfony IOL does not create an out-of-focus image that would generate halos, which may help explain the low incidence rate of photic phenomena in our study. In contrast, in the monofocal IOL group, less patients were disturbed by photic phenomena. Previous studies also established that patients who received multifocal IOLs experienced dysphotopsia more often than those with monofocal IOLs [14, 46].

In this study, the contrast sensitivity values measured with the CSV-1000 showed good levels under both photopic and mesopic conditions. Though the values decreased at high spatial frequencies, this was still within the normal range and consistent with previous reports. Ruiz-Mesa et al. [21] observed no significant differences in levels of contrast sensitivity between a group of eyes implanted with Tecnis Symfony IOLs and another group of eyes with PanOptix IOLs. de Medeiros et al. [17] showed that under photopic conditions, patients who received Tecnis Symfony IOL on one eye and a bifocal lens on the other (Tecnis® ZMB00) showed better results at low spatial frequencies. Nevertheless, it has been reported that in patients with multifocal lenses, the performance of contrast sensitivity decreases at high spatial frequencies [25, 47]. Further research is required to evaluate the contrast sensitivity of the patients with bilateral implantation of Tecnis Symfony IOLs.

According to our results, the residual refractive error after implantation of the studied EDOF IOL had a very limited impact on monocular UDVA compared to the monofocal IOL with the same platform. In all subdivision groups, the EDOF group always showed better UDVA than the monofocal IOL. It is also important to note that we found no statistically significant differences in the mean pupil sizes measured pre- and postoperatively between the EDOF and the monofocal groups. Cochener et al. [48] observed that the Tecnis Symfony lens demonstrates an excellent tolerance to unexpected refractive errors and that monocular and binocular UDVA did not greatly change, with a mere difference of 0.05 between postoperative SE of ±0.25 and ± 2.00 D. In another study, Carones et al. compared the impact of induced astigmatism on the visual acuity in four different types of multifocal lenses and observed the highest tolerance with the Symfony IOL, which retained good visual acuity (0.7, decimal scale) even with an induced astigmatism of − 1.50 D [49]. Multifocal IOLs require emmetropia as target refraction to achieve the best visual outcomes and small amounts of refractive error may considerably degrade their visual performance [3]. Thus, refractive error must be fully corrected to achieve the maximum visual potential of a multifocal IOL [4]. However, residual refractive errors can be related to a variety of factors and it is not possible to predict absolute postoperative refractive errors. Besides, residual refractive error is the main identifiable cause of blurred vision [50] and can lead to high levels of dissatisfaction [2]. Such dissatisfaction can lead to lens explantation, IOL exchange, or laser refractive surgery postoperatively [51, 52]. Regarding the postoperative SE, the Tecnis Symfony IOL showed a stable tolerance to unexpected postoperative refractive errors. Such characteristic adds an additional value to the optical property of this lens and renders it versatile for different clinical situations, which is a key factor for high satisfaction rate. Further study is yet required to assess the visual acuity tolerance to the postoperative SE of patients with bilateral implantation of Symfony IOLs.

The study has several notable limitations. First, the refractive tolerances were only tested with an EDOF and a monofocal IOL. Ideally, more than three groups (other type of diffractive-refractive, EDOF, or monofocal IOLs) would have to be included to minimize confounding factor. Additionally, the EDOF lens was targeted for emmetropia, which may result in better intermediate vision, not for near vision. Lastly, our study did not analyze the optical performance of different IOL designs on an optical bench to characterize their behavior. Future studies should address these limitations. As certain multifocal IOLs remain unknown with respect to visual acuity tolerance to postoperative refractive errors, future randomized comparative studies with a larger cohort of the Tecnis Symfony IOL and other types of multifocal IOLs should be conducted to confirm its superiority in terms of residual refractive error and visual acuity tolerance to the postoperative SE.

To summarize, the Tecnis Symfony EDOF IOL is a promising means for producing excellent visual rehabilitation in patients receiving presbyopic treatment. Although previous studies showed that near vision was better in trifocal IOLs compared to the EDOF IOLs, EDOF IOLs provided superior far and intermediate vision and high levels of postoperative visual acuity tolerance than other types of monofocal or multifocal IOLs that are currently available. To minimize the optical side effects of multifocality, EDOF IOLs represent new-generation multifocal IOLs that provide a better alternative to patients who desire a spectacle-free lifestyle postoperatively. Further studies with longer follow-up periods may help provide quantitative long-term information on the optical properties of EDOF IOLs in comparison to the currently gold standard monofocal IOL and ultimately assist surgeons in choosing the appropriate IOL design for individual patients.

## Data Availability

Not applicable.

## Abbreviations

CDVA: Corrected distance visual acuity
EDOF: Extended depth of focus
IOLs: Intraocular lenses
SE: Spherical equivalent
UDVA: Uncorrected distance visual acuity
UIVA: Uncorrected intermediate visual acuity
UNVA: Uncorrected near visual acuity
VA: Visual acuity

## References

[1] Charman WN (2014). Developments in the correction of presbyopia II: surgical approaches. Ophthalmic Physiol Opt.

[2] de Vries NE, Webers CA, Touwslager WR, Bauer NJ, de Brabander J, Berendschot TT (2011). Dissatisfaction after implantation of multifocal intraocular lenses. J Cataract Refract Surg.

[3] Macsai MS, Fontes BM (2008). Refractive enhancement following presbyopia-correcting intraocular lens implantation. Curr Opin Ophthalmol.

[4] Abdelghany AA, Alio JL (2014). Surgical options for correction of refractive error following cataract surgery. Eye Vis (Lond).

[5] Gundersen KG, Makari S, Ostenstad S, Potvin R (2016). Retreatments after multifocal intraocular lens implantation: an analysis. Clin Ophthalmol.

[6] Artal P, Manzanera S, Piers P, Weeber H (2010). Visual effect of the combined correction of spherical and longitudinal chromatic aberrations. Opt Express.

[7] Lopez-Gil N, Montes-Mico R (2007). New intraocular lens for achromatizing the human eye. J Cataract Refract Surg.

[8] Pomerance GN, Evans DW (1994). Test-retest reliability of the CSV-1000 contrast test and its relationship to glaucoma therapy. Invest Ophthalmol Vis Sci.

[9] Carballo-Alvarez J, Vazquez-Molini JM, Sanz-Fernandez JC, Garcia-Bella J, Polo V, Garcia-Feijoo J (2015). Visual outcomes after bilateral trifocal diffractive intraocular lens implantation. BMC Ophthalmol.

[10] Denoyer A, Le Lez ML, Majzoub S, Pisella PJ (2007). Quality of vision after cataract surgery after Tecnis Z9000 intraocular lens implantation: effect of contrast sensitivity and wavefront aberration improvements on the quality of daily vision. J Cataract Refract Surg.

[11] Alfonso JF, Fernandez-Vega L, Puchades C, Montes-Mico R (2010). Intermediate visual function with different multifocal intraocular lens models. J Cataract Refract Surg.

[12] Anton A, Bohringer D, Bach M, Reinhard T, Birnbaum F (2014). Contrast sensitivity with bifocal intraocular lenses is halved, as measured with the Freiburg vision test (FrACT), yet patients are happy. Graefes Arch Clin Exp Ophthalmol.

[13] Calladine D, Evans JR, Shah S, Leyland M. Multifocal versus monofocal intraocular lenses after cataract extraction. Cochrane Database Syst Rev. 2012;12(9):CD003169. 10.1002/14651858.CD003169.pub3 22972061

[14] Cillino S, Casuccio A, Di Pace F, Morreale R, Pillitteri F, Cillino G (2008). One-year outcomes with new-generation multifocal intraocular lenses. Ophthalmology..

[15] Owsley C, Stalvey BT, Wells J, Sloane ME, McGwin G (2001). Visual risk factors for crash involvement in older drivers with cataract. Arch Ophthalmol.

[16] Vryghem JC, Heireman S (2013). Visual performance after the implantation of a new trifocal intraocular lens. Clin Ophthalmol.

[17] de Medeiros AL, de Araujo Rolim AG, Motta AFP, Ventura BV, Vilar C, Chaves M (2017). Comparison of visual outcomes after bilateral implantation of a diffractive trifocal intraocular lens and blended implantation of an extended depth of focus intraocular lens with a diffractive bifocal intraocular lens. Clin Ophthalmol.

[18] Ferreira TB, Pinheiro J, Zabala L, Ribeiro FJ (2018). Comparative analysis of clinical outcomes of a monofocal and an extended-range-of-vision intraocular lens in eyes with previous myopic laser in situ keratomileusis. J Cataract Refract Surg.

[19] Monaco G, Gari M, Di Censo F, Poscia A, Ruggi G, Scialdone A (2017). Visual performance after bilateral implantation of 2 new presbyopia-correcting intraocular lenses: trifocal versus extended range of vision. J Cataract Refract Surg.

[20] Ruiz-Mesa R, Abengozar-Vela A, Aramburu A, Ruiz-Santos M (2017). Comparison of visual outcomes after bilateral implantation of extended range of vision and trifocal intraocular lenses. Eur J Ophthalmol.

[21] Ruiz-Mesa R, Abengozar-Vela A, Ruiz-Santos M (2018). A comparative study of the visual outcomes between a new trifocal and an extended depth of focus intraocular lens. Eur J Ophthalmol.

[22] Hogarty DT, Russell DJ, Ward BM, Dewhurst N, Burt P (2018). Comparing visual acuity, range of vision and spectacle independence in the extended range of vision and monofocal intraocular lens. Clin Exp Ophthalmol.

[23] Cochener B, Boutillier G, Lamard M, Auberger-Zagnoli C (2018). A comparative evaluation of a new generation of diffractive trifocal and extended depth of focus intraocular lenses. J Refract Surg.

[24] Alfonso JF, Puchades C, Fernandez-Vega L, Montes-Mico R, Valcarcel B, Ferrer-Blasco T (2009). Visual acuity comparison of 2 models of bifocal aspheric intraocular lenses. J Cataract Refract Surg.

[25] Alio JL, Montalban R, Pena-Garcia P, Soria FA, Vega-Estrada A (2013). Visual outcomes of a trifocal aspheric diffractive intraocular lens with microincision cataract surgery. J Refract Surg.

[26] Alio JL, Pinero DP, Plaza-Puche AB, Chan MJ (2011). Visual outcomes and optical performance of a monofocal intraocular lens and a new-generation multifocal intraocular lens. J Cataract Refract Surg.

[27] Alio JL, Plaza-Puche AB, Pinero DP, Amparo F, Jimenez R, Rodriguez-Prats JL (2011). Optical analysis, reading performance, and quality-of-life evaluation after implantation of a diffractive multifocal intraocular lens. J Cataract Refract Surg.

[28] Cillino G, Casuccio A, Pasti M, Bono V, Mencucci R, Cillino S (2014). Working-age cataract patients: visual results, reading performance, and quality of life with three diffractive multifocal intraocular lenses. Ophthalmology..

[29] Cochener B, Vryghem J, Rozot P, Lesieur G, Chevalier JP, Henry JM (2014). Clinical outcomes with a trifocal intraocular lens: a multicenter study. J Refract Surg.

[30] Jonker SM, Bauer NJ, Makhotkina NY, Berendschot TT, van den Biggelaar FJ, Nuijts RM (2015). Comparison of a trifocal intraocular lens with a +3.0 D bifocal IOL: results of a prospective randomized clinical trial. J Cataract Refract Surg.

[31] Kohnen T, Titke C, Bohm M (2016). Trifocal intraocular lens implantation to treat visual demands in various distances following lens removal. Am J Ophthalmol.

[32] Kretz FT, Gerl M, Gerl R, Muller M, Auffarth GU, Group ZKBS (2015). Clinical evaluation of a new pupil independent diffractive multifocal intraocular lens with a +2.75 D near addition: a European multicentre study. Br J Ophthalmol.

[33] Law EM, Aggarwal RK, Kasaby H (2014). Clinical outcomes with a new trifocal intraocular lens. Eur J Ophthalmol.

[34] Lubinski W, Gronkowska-Serafin J, Podboraczynska-Jodko K (2014). Clinical outcomes after cataract surgery with implantation of the Tecnis ZMB00 multifocal intraocular lens. Med Sci Monit.

[35] Mojzis P, Kukuckova L, Majerova K, Liehneova K, Pinero DP (2014). Comparative analysis of the visual performance after cataract surgery with implantation of a bifocal or trifocal diffractive IOL. J Refract Surg.

[36] Mojzis P, Pena-Garcia P, Liehneova I, Ziak P, Alio JL (2014). Outcomes of a new diffractive trifocal intraocular lens. J Cataract Refract Surg.

[37] Ramon ML, Pinero DP, Perez-Cambrodi RJ (2012). Correlation of visual performance with quality of life and intraocular aberrometric profile in patients implanted with rotationally asymmetric multifocal IOLs. J Refract Surg.

[38] Schmickler S, Bautista CP, Goes F, Shah S, Wolffsohn JS (2013). Clinical evaluation of a multifocal aspheric diffractive intraocular lens. Br J Ophthalmol.

[39] Sheppard AL, Shah S, Bhatt U, Bhogal G, Wolffsohn JS (2013). Visual outcomes and subjective experience after bilateral implantation of a new diffractive trifocal intraocular lens. J Cataract Refract Surg.

[40] Gundersen KG (2018). Rotational stability and visual performance 3 months after bilateral implantation of a new toric extended range of vision intraocular lens. Clin Ophthalmol.

[41] Marques EF, Ferreira TB, Simoes P (2016). Visual performance and rotational stability of a multifocal toric intraocular lens. J Refract Surg.

[42] Pedrotti E, Bruni E, Bonacci E, Badalamenti R, Mastropasqua R, Marchini G (2016). Comparative analysis of the clinical outcomes with a monofocal and an extended range of vision intraocular lens. J Refract Surg.

[43] Cochener B, Concerto SG (2016). Clinical outcomes of a new extended range of vision intraocular lens: international multicenter Concerto study. J Cataract Refract Surg.

[44] Alba-Bueno F, Vega F, Millan MS (2014). Halos and multifocal intraocular lenses: origin and interpretation. Arch Soc Esp Oftalmol.

[45] Esteve-Taboada JJ, Dominguez-Vicent A, Del Aguila-Carrasco AJ, Ferrer-Blasco T, Montes-Mico R (2015). Effect of large apertures on the optical quality of three multifocal lenses. J Refract Surg.

[46] Chiam PJ, Chan JH, Aggarwal RK, Kasaby S (2006). ReSTOR intraocular lens implantation in cataract surgery: quality of vision. J Cataract Refract Surg.

[47] Wang M, Corpuz CC, Fujiwara M, Tomita M (2014). Visual and optical performance of diffractive multifocal intraocular lenses with different haptic designs: 6 month follow-up. Clin Ophthalmol.

[48] Cochener B (2017). Tecnis symfony intraocular lens with a “sweet spot” for tolerance to postoperative residual refractive errors. Open J Ophthalmol.

[49] Carones F (2017). Residual astigmatism threshold and patient satisfaction with bifocal, trifocal, and extended range of vision intraocular lenses (IOLs). Open J Ophthalmol.

[50] Gibbons A, Ali TK, Waren DP, Donaldson KE (2016). Causes and correction of dissatisfaction after implantation of presbyopia-correcting intraocular lenses. Clin Ophthalmol.

[51] Kim EJ (2017). Sajjad a, Montes de Oca I, Koch DD, Wang L, Weikert MP, et al. refractive outcomes after multifocal intraocular lens exchange. J Cataract Refract Surg.

[52] Santhiago MR, Ventura BV, Ghanem RC, Kara-Junior N, Moraes HV, Ghanem E (2016). Predictability and vector analysis of laser in situ keratomileusis for residual errors in eyes implanted with different multifocal intraocular lenses. Cornea..

